# Innate Immune Response Regulation by the Human *RNASET2* Tumor Suppressor Gene

**DOI:** 10.3389/fimmu.2019.02587

**Published:** 2019-11-05

**Authors:** Francesco Acquati, Lorenzo Mortara, Annarosaria De Vito, Denisa Baci, Adriana Albini, Marco Cippitelli, Roberto Taramelli, Douglas M. Noonan

**Affiliations:** ^1^Human Genetics Laboratory, Department of Biotechnology and Molecular Sciences, University of Insubria, Varese, Italy; ^2^Immunology and General Pathology Laboratory, Department of Biotechnology and Life Sciences, University of Insubria, Varese, Italy; ^3^School of Medicine and Surgery, University of Milano-Bicocca, Monza, Italy; ^4^Scientific and Technology Pole, IRCCS MultiMedica, Milan, Italy; ^5^Department of Molecular Medicine, Faculty of Pharmacy and Medicine, University La Sapienza, Rome, Italy

**Keywords:** T2 RNases, innate immune response, tumor suppression, stress response, tumor microenvironment, targeting immunotherapy

## Abstract

The link between cancer development or progression and immune system dysregulation has long been established. Virtually every cell type belonging to both the innate and adaptive immune system has been reported to be involved in a complex interplay that might culminate into either a pro- or anti-tumorigenic response. Among the cellular components of the innate immune system, cells belonging to the monocyte/macrophage lineage have been consistently shown to play a key role in the tumorigenic process. The most advanced human tumors are reported to be strongly infiltrated with Tumor-Associated Macrophages (TAMs) endowed with the ability to contribute to tumor growth and dissemination. However, given their widely acknowledged functional plasticity, macrophages can display anti-tumor properties as well. Based on these premises, experimental approaches to promote the *in vivo* macrophage shift from pro-tumor to anti-tumor phenotype represent one of the most promising research field aimed at developing immune system-mediated tumor suppressive therapies. In this context, the human *RNASET2* oncosuppressor gene has emerged as a potential tool for macrophage-mediated tumor suppression. A growing body of experimental evidence has been reported to suggest a role for this gene in the regulation of macrophage activity in both *in vitro* and *in vivo* experimental models. Moreover, several recent reports suggest a role for this gene in a broad range of cell types involved in immune response, pointing at *RNASET2* as a putative regulator of several functional features within the immune system.

## Introduction

The innate immune system represents an evolutionary conserved host defense tool, with many key features being shared between plants, invertebrates, and vertebrates (1).

However, dysfunction of this defense system is also involved in a wide range of pathologies, which include cancer and autoimmune disease in humans (2, 3).

In cancer, key cellular components of the innate immune system undergo a significant alteration in their effector functions, whose final result is the expression of tolerant or pro-tumor functions (4). By contrast, autoimmune diseases are usually associated with an abnormal, excessive response of CD4^+^ T helper (Th) cell subsets in cooperation with myeloid innate immune cells (5, 6). Despite recent significant progresses have been achieved in understanding cancer biology, the diagnosis and treatment of cancer still represent one the leading cause of death in western countries, whereas autoimmune diseases still strike millions of people worldwide. In cancer, tumor cells make themselves invisible to the adaptive immune system, by up-regulating self-defense mechanisms which promote immunological self-tolerance (7). The discovery of immune check-point molecules that limit autoimmunity, and their blockade in cancer treatment, has been recently exploited as an anti-cancer therapeutic approach (8, 9).

Among the cellular components of the innate immune system, macrophages represent key effector cells (10). These cells display direct effector roles in the control of pathogen infections and cancer cells destruction, regulate the inflammatory response and modulate the adaptive immune cells. However, their role in cancer control is controversial, since they can also carry out roles whose final effect is to promote, rather than hinder, cancer growth (11).

These alternative functions reside in the ability of macrophages to display phenotypes endowed with specific functional roles, as exemplified by the two opposite polarization states (the M1-like anti-tumor and M2-like pro-tumor phenotypes, respectively) that macrophages can experience in response to different microenvironmental stimuli (12, 13). However, it has been recently established that macrophage polarization in several physiologic and pathologic conditions actually represents a continuum, in which these cells display a spectrum of distinct polarization states that do not fit to the oversimplified M1/M2 classification (14).

Reflecting their plasticity, within the tumor microenvironment (TME) macrophages acquiring distinct phenotypes and functions (resulting in the attenuation of their antitumor activity and induction of tumor-supporting functions) have been defined as tumor-associated macrophages (TAMs) with M2-like features. TAMs represent a mixed cell population with heterogeneous phenotypes and functions, which includes resident macrophages, infiltrating blood monocytes, and monocyte-related myeloid-derived suppressor cells, known to be involved in tumor initiation, growth, angiogenesis, metastasis, immunosuppression, cancer-related inflammation, and resistance to therapy (15).

Besides the widely acknowledged role of macrophages in the control of cancer growth *in vivo*, other components of the TME also strongly affect cancer progression, such as extracellular matrix (ECM) molecules, fibroblasts, endothelial cells, and other types of innate and adaptive immune cells (16).

For instance, natural killer (NK) cells are known to be altered under hypoxic conditions (a typical stress condition experienced by cancer cells) by assuming a uterine/decidua-like NK (dNK) cell phenotype (17) which display low cytotoxicity and is involved in angiogenesis and blastocyst implantation (18–20). NK cells are effector lymphocytes of the innate immunity endowed with cytotoxic activity and Th1 cytokine production (17). However, in cancer patients NK cells display very low cytotoxicity (21, 22) and a pro-angiogenic phenotype (23–26).

The involvement of the innate immune system in the control of cancer growth thus entails a complex crosstalk between most of its cellular components, whose interplay is just beginning to be defined. In fact, escaping the immune surveillance is now widely accepted as the seventh hallmark of cancer (27) and a widely pursued task in current cancer research is aimed at “reinstructing” the immune system to restore or enhance its anti-cancer activity (13).

Elucidating the roles and mechanisms of action of the molecular effectors within the TME which impact on the balance between the pro- and anti-tumor roles of the innate immune system thus represents a key topic in current cancer research, since it may open new opportunities for therapeutic interventions. In this context, several members of the T2 ribonuclease enzyme family have recently emerged as potential key players in innate immunity-mediated cancer growth control, by acting as stress-response, “alarmin”-like tumor suppressor genes.

## T2 Ribonucleases: An Emerging Family of Evolutionarily Conserved, Highly Pleiotropic Proteins

Ribonucleases (RNases) represent RNA-processing or degrading enzymes found in almost all organisms. They participate in many key cellular functions, such as DNA replication, control of gene expression, extracellular signaling and host defense (28).

Being RNAs involved in key biological processes (29), proteins affecting RNA turnover have been thoroughly investigated to better understand their role in basic cellular processes, such as cell proliferation, differentiation, apoptosis, and migration. Of note, dysregulation of these biological processes are known to be involved in cancer development.

Among ribonucleases that hydrolyze single-stranded RNA (30), transferase-type represent an important subclass, epitomized by the extensively investigated RNase A protein family (31). A key feature of these enzymes is their secretion in the extracellular milieu or their localization in several subcellular structures. Despite these ribonucleases have been classified in several ways, in broad terms they are classified as alkaline ribonucleases (T1 and A families) and acid ribonucleases (T2 family) (32).

**Figure 1:**
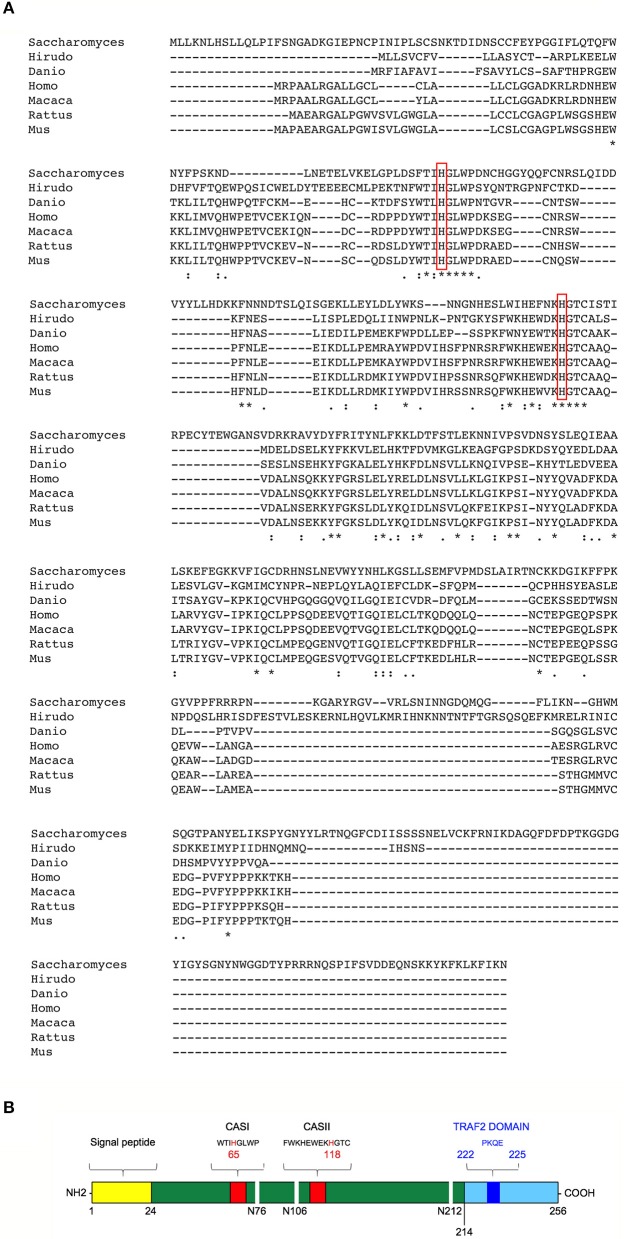
Structural features and evolutionary conservation of T2 RNase proteins. **(A)** A Clustal Omega alignment of several members of the T2 RNase family, showing the wide evolutionary conservation of this enzymes. RNA cleavage by T2 RNases is mediated by histidine residues (red boxes) embedded into two highly conserved motifs dubbed CAS I and CAS II. The species included in the alignment are *Saccharomices cerevisiae, Hirudo verbana, Danio rerio, Homo sapiens, Macaca mulatta, Rattus norvegicus*, and *Mus musculus*. **(B)** Structure of human RNASET2. The RNASET2 primary sequence includes 256 aminoacid residues, with a predicted molecular weight of about 30 kDa. The core enzyme is colored in dark green in the figure. Among the protein's structural features, a 24-residues long signal peptide for secretion at the N-terminal (yellow bar) and the two canonical CAS sites (CAS I/II, red bars) responsible for the enzyme's catalytic activity (bearing a highly conserved key histidine residue at position 65 and 118, respectively) are shown in the figure. Three N-glycosylation sites (white horizontal lines), which increase the molecular weight of the native protein of about 6 kDa, are also shown (34). Finally, a putative TRAF-2 binding site (dark blue bar) was predicted in the C-terminal part of RNASET2 (light blue bar, starting form residue 214), which is less evolutionary conserved throughout evolution and has been suggested to be comprise a highly disordered loop (35). Within the cell, the RNASET2 protein is present in three forms of different sizes, namely 36, 31, and 27 kDa (34). The 36 kDa isoform represents the full-length and secreted form, which is easily detected in cell culture supernatants from RNASET2-expressing cells, whereas the other two isoforms represent intracellular protein isoforms originating from proteolytic cleavage of the full-length protein **(B)**.

T2 ribonucleases were originally classified by their similarity to the first acid ribonuclease purified from *Aspergillus oryzae* (33). T2 ribonucleases can be distinguished from A and T1 family members based on their preferential acidic pH for optimal catalytic activity and their impressively wide evolutionary conservation (unlike T1 and A ribonucleases, T2 ribonucleases have been widely reported among *taxa*) (Figure 1A) (36). Such striking pattern of evolutionary conservation suggests a very ancient and key role for this class of ribonucleases. All members of the T2 ribonucleases family are also characterized by two characteristic catalytic sites (CAS) I and II motifs, endowed with the catalytic function (Figure 1B).

The extremely wide evolutionary conservation of T2 ribonucleases is coupled to their involvement in several physiological functions, ranging from scavenging phosphate for nutritional needs to neural development, prevention of self-pollination in plants, cell cytotoxicity, modulation of the cytoskeleton, angiogenesis, and stress response (36).

Noteworthy, many of these biological processes are implicated in cancer development. For instance, the T2 ribonucleases from *Aspergillus niger* has been reported to display both anticarcinogenic and antiangiogenic properties (37, 38).

Moreover, like several vertebrate ribonucleases A family members (39), T2 ribonucleases from different species have been recently reported to be involved in immune response modulation, another biological process tightly linked to cancer growth control. For instance, the RNASET2 protein secreted by the parasite *Schistosoma mansonii* has been reported to prime host dendritic cells to trigger a Th2 polarization of CD4^+^ T lymphocytes during infection (40–42), although the reported effect of the *Schistosoma* T2 RNase on the host innate immune response was different from that later described in mammalian experimental models, being dendritic cells rather than macrophages the main target of the protein. This apparently different roles of T2 ribonuclease family members might reflect the evolutionary distant experimental models used (i.e., a trematode parasite and mammalian species).

Moreover, transcriptional profiling of human MEC-1 cells-derived leukemic tumors developing *in vivo* in a murine experimental model where host macrophages were depleted showed a marked downregulation of human *RNASET2* expression in actively growing tumors only (43). The last report suggested an oncosuppressive role for human *RNASET2* mediated by the involvement of the host innate immune system.

## Human RNASET2: An Alarmin-Like Tumor Suppressor Gene Acting on Cells From the Monocyte/Macrophage Lineage

The *RNASET2* gene represents the only human member of the T2 extracellular ribonucleases family and has been mapped on human 6q27, a region which has been frequently found to be rearranged in a wide range of cancers (44–50). The RNASET2 protein includes 256 aminoacid residues, encoding a signal peptide at the N-terminal and the two canonical I/II catalytic sites (Figure 1B) (34). A putative Tumor necrosis factor Receptor-Associated Factor-2 (TRAF-2) binding site was also predicted in the C-terminal part and has been suggested to play a role in RNASET2-mediated apoptosis in both human melanocytes and keratinocytes (35). T2 ribonucleases are normally extracellular proteins, but intracellular isoforms (34) have also been detected in the secretory pathway, lysosomes, mitochondria and processing bodies, cytoplasmic ribonucleoprotein (RNP) granules primarily composed of translationally repressed mRNAs and proteins related to mRNA decay (51–53).

Being localized in a chromosomal region that represents a common target for rearrangements in a wide spectrum of cancer types, the putative role of *RNASET2* as a tumor suppressor gene has long been investigated.

Our group initially chose human ovarian carcinoma as an experimental model to test the role of *RNASET2* as a tumor suppressor gene. Indeed, this gene shows an expression pattern which is compatible with a role in ovarian cancer, being expressed in ovarian and fallopian tube surface epithelia, the structures from which most ovarian carcinomas are thought to arise (54). Interestingly, we showed the *RNASET2* gene to be frequently downregulated in both ovarian cancer-derived cell lines and tumor samples (55).

To better define the function of *RNASET2*, both ovarian carcinoma and malignant melanoma-derived human cell lines were used for *in vivo* xenograft assays carried out in nude mice. Strikingly, *RNASET2*-overexpressing clones derived from both cell lines displayed a marked suppression of their tumorigenic potential *in vivo* (56, 57).

Tumors derived from human cancer cells overexpressing a catalytically inactive RNASET2 protein were equally suppressed in their *in vivo* growth rate when compared to their wildtype RNASET2-expressing counterpart, suggesting that *RNASET2*-mediated tumor suppression is independent from its ribonuclease activity (57). The latter was not a completely unexpected finding, since other members of the T2 ribonuclease family are known to carry out a particular biological process independently of their catalytic activity (36).

A detailed histological survey showed that xenograft-derived suppressed tumors overexpressing RNASET2 were strongly infiltrated by murine stromal cells belonging to the M1 subclass of macrophages, which are known to display a marked anti-tumorigenic role (57). These data were confirmed by a further *in vivo* xenograft-based assay, where *in vivo* depletion of host macrophages largely restored the tumorigenic potential of *RNASET2-*overexpressing human ovarian cancer cells (57). These data strongly pointed at the monocyte/macrophage cell lineage as a key component of *RNASET2-*mediated tumor suppression.

The crucial role of host macrophages was confirmed in an independent xenograft-based model, whereby knock-down of endogenously expressed *RNASET2* in human OVCAR3 ovarian cancer cells was associated with a marked increase in their growth rate *in vivo*, coupled with a significant decrease of M1-polarized macrophage infiltration (58). Moreover, gene expression analysis in two human cancer types (ovarian cancer and chronic lymphocytic leukemia) unveiled a gradual decrease of *RNASET2* gene expression with increasing stage or grade (which is an expected pattern for an oncosuppressor gene) (59). However, both cancer types, actually showed a marked *upregulation* of RNASET2 expression at early stages when compared to the healthy tissue, followed by a gradual decrease in advanced stages.

**Figure 2:**
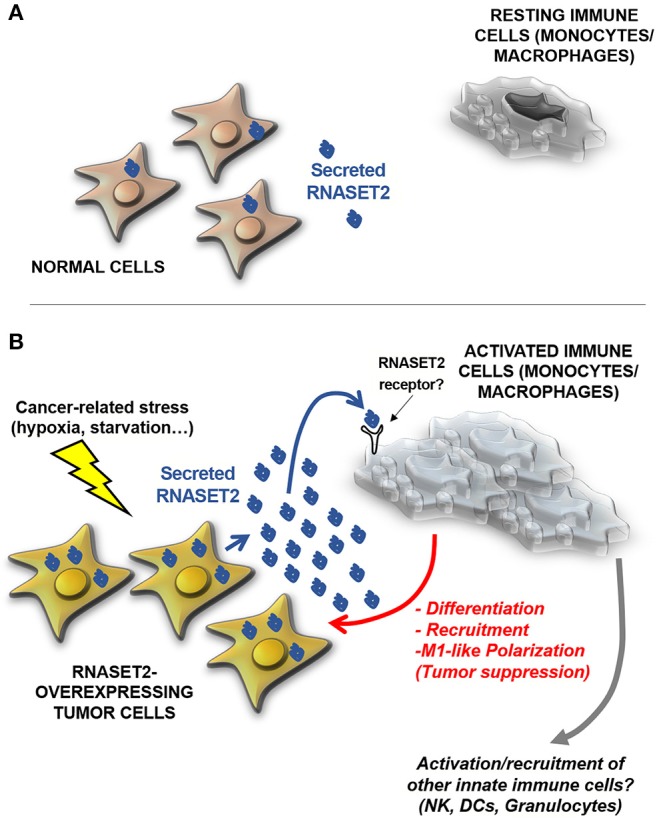
A model for human RNASET2-mediated tumor suppression. **(A)** In physiological contexts, most human cells express low or undetectable RNASET2 levels (https://www.ncbi.nlm.nih.gov/gene/8635), with the notable exception of spleen, lymph nodes and colon, three tissues highly involved in immune system function. This ubiquitous “baseline” RNASET2 expression can be related to the execution of intracellular or extracellular roles (some yet-to-be defined) possibly mediated by its catalytic activity. **(B)** When cells are locally exposed to a wide range of stresses, some of which are typically experienced by cancer cells (such as hypoxia, oxidative stress or nutritional starvation), they activate a “danger-response” program which involves, besides the activation of several endogenous stress response pathways, a massive increase in expression and secretion of RNASET2, which acts as an alarmin-like molecule to engage cells belonging to the innate immune system (mostly macrophages, but possibly other cellular components such as natural killer (NKs), dendritic cells (DCs) and granulocytes) to coordinate an immune-response-mediated tumor suppressive response. As previously described for several biological processes mediated by T2 RNases, the catalytic activity of the RNASET2 is not required to trigger this marked immune system-mediated response. Strikingly, the functional crosstalk between RNASET2 and cellular effectors of the innate immune system has been reported in several evolutionary distant species, suggesting an ancient key role of T2 RNases in immune-response mediated host defense.

These data strongly pointed at a *non-cell autonomous* oncosuppressive role of RNASET2, by which cancer cells secreting high levels of this protein might send a sort of “alarm” message to monocytes/macrophages in order to promote their oncosuppressive activity by means of their active recruitment, activation, and polarization (Figure 2). According to this hypothesis, RNASET2 might represent a novel member of the “alarmin” family, molecules passively released by necrotic cells or actively secreted by epithelial or immune cells in order to signal to the innate immune system the occurrence of dangerous events (60, 61).

In keeping with the alarmin hypothesis, overexpression in human ovarian cancer cells of an engineered RNASET2 protein bearing an endoplasmic reticulum retention signal (to prevent protein secretion) largely restored the ability of these cells to form fast-growing tumors *in vivo*, thus demonstrating the key role of extracellular RNASET2 in tumor suppression (62). A role in innate immune system modulation has been reported for other extracellular ribonucleases, such as some human ribonuclease A family members (39) and, significantly, a T2 ribonuclease secreted by the *Schistosoma mansonii* parasite's eggs during mammalian infection (40, 41).

Furthermore, alarmins are known to work as stress response proteins and, accordingly, several members of the T2 ribonuclease family are known to act as stress response genes in several species (51, 62–64).

As long as human RNASET2 is concerned, its expression and extracellular secretion are markedly increased both *in vitro* and *in vivo* under a wide range of stressful conditions (35, 62, 65, 66), among which hypoxia and nutritional starvation, two stressful conditions typically experienced by early-stage cancer cells.

Gene expression analysis based on publicly available datasets indicates that *RNASET2* is also expressed in Multiple Myeloma (MM) cells (http://www.humanmine.org-RNASET2). Interestingly, although expression levels *RNASET2* in MM patients does not change much from its precancerous Monoclonal Gammopathy of Undetermined Significance stage, recent evidence suggest that this gene is significantly regulated by epigenetic modifications, as observed in *t*_(4;14)+_ myeloma cells overexpressing the histone methyltransferase MMSET, a driving factor in the pathogenesis of this subtype of myeloma.

Investigations of the T2 ribonuclease from *Aspegillus niger* (ACTIBIND) based on xenograft models have confirmed the strong, non cell-autonomous *in vivo* tumor suppressive activity for this class of proteins (37, 38).

The functional nature of the crosstalk between RNASET2 and cells from the monocyte/macrophage lineage was also recently investigated. Strikingly, human recombinant RNASET2 was shown to act as a potent chemokine for cells belonging to the monocyte/macrophage lineage (58), in keeping with the previous *in vivo* data (57, 58).

Furthermore, knock-down of *endogenous RNASET2* expression in the human promonocytic THP1 cell line model was shown to affect the polarization pattern of differentiated THP-1-derived macrophages by promoting a shift form the anti-tumor M1 to the pro-tumor M2 state (67). These data provide a further support to the previously reported recruitment of M1-polarized macrophages in RNASET2-overexpressing tumor xenografts and point at RNASET2 as an alarmin-like molecule.

Given the extreme evolutionary conservation of T2 ribonucleases, their role as innate immune system modulators has been recently investigated in non-vertebrate experimental models. Strikingly, recombinant RNASET2 injection in the body wall of the invertebrate medicinal leech *Hirudo verbana* triggered a massive recruitment of AIF1^+^ host macrophages into the injected area, again supporting the role of RNASET2 as a chemoattractant molecule for monocyte/macrophages (68).

The recruited macrophages were shown to be functionally activated as phagocytic cells and to actively express their endogenous *RNASET2* gene, suggesting the occurrence of a putative RNASET2-mediated positive feedback in these cells (68). The confirmation of a functional crosstalk between a T2 ribonuclease and tissue macrophages in evolutionarily distant *taxa* is of key relevance, since it suggests a very ancient and conserved role for T2 ribonucleases in host defense.

## Is the Crosstalk Between Human Rnaset2 and the Immune System Wider Than Expected? Hints From Recent Experimental Data

In recent years, further investigations led to the discovery of the molecular pathways by which T2 ribonuclease family members carry out their oncosuppressive role. Therefore, depending on the adopted experimental system, T2 ribonucleases have been reported to control key cellular processes such as angiogenesis, apoptosis, cytoskeletal rearrangements, cell invasion, and innate immune cells activation or polarization (35, 38, 62, 63, 67, 69). Based on these data, T2 ribonucleases are currently considered highly pleiotropic proteins endowed with an ancient, evolutionary conserved role related to stress response and host defense.

In this context, granulocytes represent further key effector cells of the innate immune system. Strikingly, our recent investigations in the medicinal leech showed endogenous RNASET2 overexpression in these cells following lipopolysaccharide injection into the body wall (70) and the RNASET2 protein was detected in the granules of these cells, suggesting a further role in innate immunity-mediated host defense.

Taken together, these data make *RNASET2* a strong candidate gene for innate immune response-mediated control of cancer growth, by means of a complex multicellular network involving other cells besides tissue macrophages.

Finally, a new piece in the puzzle has been recently added by the results of several genome-wide association studies (GWAS), where a few SNPs nearby the *RNASET2* gene have been strongly associated with the risk for several human autoimmune diseases, such as Grave's disease, vitiligo, Crohn's disease, rheumatoid arthritis, and type I diabetes (71–75). Some of these polymorphisms were shown to modulate RNASET2 expression (76), again suggesting a key a role for RNASET2 in host immune response regulation.

Given the known functional link between autoimmune disease and cells from the innate immune systems (77), these recent observations further support a pleiotropic and widespread role for T2 ribonucleases in immune system-mediated host defense mechanisms.

## Conclusion

The *RNASET2* gene is the only human member of this extracellular ribonuclease gene family. Unlike other ribonucleases, T2 ribonucleases have been discovered in most organisms and are mostly related to stress response and host defense. Significantly, the roles assigned to T2 ribonucleases are often mediated by biological processes tightly linked to cancer development (36).

Despite some of these processes apparently suggest a *cell-autonomous* oncosuppressor role for T2 ribonucleases, recent experimental data point at the occurrence of a functional crosstalk between members of this extracellular protein family and the tumor microenvironment as well.

Interestingly, establishing this crosstalk represents a key step in T2 ribonuclease-mediated tumor suppression in some *in vivo* experimental models. In particular, T2 ribonucleases from evolutionary distant species such as *Homo sapiens, Schistosoma mansonii* and *Hirudo verbana* all share a common ability to functionally interact with and modulate one or more cellular effectors of the innate immune system. These data suggest that modulation of the immune system by T2 ribonucleases represent a very ancient feature aimed at coordinating an effective host defense response.

Beside this, in light of the growing attention for anticancer immunotherapy approaches (78), RNASET2-mediated regulation of the immune system might suggest an innovative approach in clinical oncology, based on the use of recombinant RNASET2 protein as a wide-range, pleiotropically acting antitumor drug, acting to fight cancer cells at both *cell-autonomous*, and *non cell-autonomous* levels.

Finally, the recent results from a number of GWAS, pointing at human *RNASET2* gene polymorphisms as a risk factor for several autoimmune diseases, add further support to the notion of a complex and widespread involvement of this protein in immune system modulation, by indicating at the same time a novel target for drug development for this group of devastating human diseases.

## Author Contributions

FA, LM, AD, DB, AA, MC, RT, and DN contributed to this work by planning, organizing, writing, revising, and assembling the manuscript and by preparing the figures.

### Conflict of Interest

The authors declare that the research was conducted in the absence of any commercial or financial relationships that could be construed as a potential conflict of interest.

## Abbreviations

dNK: decidua-like Natural Killer cells
ECM: Extracellular Matrix
GWAS: Genome wide association studies
LPS: lipopolysaccharide
MM: Multiple Myeloma
MMSET: Multiple myeloma set domain
NK: Natural Killer cells
SNPs: Single nucleotide polymorphisms
TAMs: Tumor-associated macrophages
TME: Tumor microenvironment
TRAF2: Tumor necrosis factor receptor-associated factor 2.
